# A cross-sectional survey on the seroprevalence of dengue fever in febrile patients attending health facilities in Cross River State, Nigeria

**DOI:** 10.1371/journal.pone.0215143

**Published:** 2019-04-22

**Authors:** Akaninyene A. Otu, Ubong A. Udoh, Okokon I. Ita, Joseph Paul Hicks, William O. Egbe, John Walley

**Affiliations:** 1 Department of Internal Medicine, University of Calabar, Calabar, Cross River State, Nigeria; 2 Foundation for Healthcare Innovation and Development (FHIND) Calabar, Cross River State, Nigeria; 3 Department of Medical Microbiology and Parasitology, University of Calabar, Calabar, Cross River State, Nigeria; 4 Nuffield Centre for International Health and Development, University of Leeds, Leeds, United Kingdom; KEMRI Wellcome Trust Research Programme, KENYA

## Abstract

**Background:**

In Nigeria, recent reports suggest that dengue viruses could be a major cause of acute fevers. We sought to make a cross-sectional estimate of the prevalence of current and previous dengue infections in patients presenting with fever to healthcare centres in Cross River State Nigeria.

**Methodology/Principal findings:**

This cross-sectional health facility survey recruited persons with temperature ≥38°C. Dengue virus immunoglobulin M (IgM)/immunoglobulin G (IgG) antibody testing using *Onsite* Duo dengue Ag-IgG/IgM lateral flow immunoassay cassettes was done. Samples which tested positive were further confirmed using the *RecombiLISA* dengue IgM and IgG enzyme linked immunosorbent assay kits and classified into primary and secondary dengue infection. Malaria testing was carried out using microscopy. Between 4 January 2017 and 24 August 2017 a total of 420 participants were sampled across 11 health centres. The mean age was 34 (range = 1–99), 63% were female, 49% reported sleeping under a treated mosquito net in the past week and 44% reported taking an antimalarial prior to seeking care. The mean number of days fever was present prior to seeking care was 8, and many of the participants presented with symptoms indicative of respiratory or urinary tract infections. Testing indicated that 6% (95% CI: 2, 13; n = 24) had either a primary or secondary dengue infection with or without co-existing malaria, while 4% (95% CI: 2, 9; n = 16) had either a primary or secondary dengue infection without co-existing malaria. 52% (95% CI: 46, 58; n = 218) had a malaria infection with or without any dengue infection, and 50% (95% CI: 44, 57; n = 210) had a malaria infection without any dengue infection.

**Conclusion:**

Our study confirms the presence of dengue at not insignificant levels in patients attending health centres with fever in this south eastern province of Nigeria. These data highlight the danger of the common presumption in this setting that fever is due to malaria. Surveillance for dengue is vital in this setting.

## Introduction

Dengue is the most important arboviral infection of humans caused by four dengue virus serotypes, namely dengue virus 1,2,3, and 4 (DENV 1–4), which belong to the *Flaviviridae* family [1]. On a global scale, the sharp increase in prevalence of dengue recorded in recent decades has caused it to be regarded as a major international public health concern. With an estimated annual incidence of 390 million cases [2], dengue poses a risk to 2.5–3.6 billion people [3] annually in over 125 endemic countries and has a case fatality rate exceeding 5% in some areas [4]. The high morbidity and economic impact of dengue are well understood in many tropical countries across Asia and Latin America [5,6]. However, across Africa the burden of dengue remains very poorly documented, despite serologic evidence indicating DENV infections are present in several countries [7,8]. In Nigeria recent reports suggest that DENV could be a major cause of acute fevers [9], although many people presenting with fever to health facilities get treated with an antimalarial without confirmatory tests despite the overlap in symptoms between malaria and dengue. The dengue mosquito vectors (principally *Aedes aegypti* and *Ae*. *albopictus*) are known to be well established [4], and serologic evidence indicates the presence of DENV infections in some cities [1,10–13]. However, evidence on the prevalence of DENV infections in Nigeria from more robust and generalisable surveys is lacking.

It is clearly important that the burden of dengue be accurately defined, ideally across relevant spatial scales for public health planning. This is particularly important given that arboviral surveillance programmes are not yet well established in Nigeria. We therefore sought to make a cross-sectional estimate of the prevalence of current and previous DENV infections in patients presenting with fever to health-care centres across Cross River State, Nigeria, and to understand the risk factors associated with DENV infection.

## Methods

### Study setting

In Cross River State there are 576 primary health-care centres (PHCs) and 157 secondary health-care centres, distributed across eighteen local government areas (LGAs), covering a population of approximately 3,866,300 [14]. These PHCs and secondary health-care centres are evenly divided across the three senatorial districts within the state which are Southern, Central and Northern senatorial districts. PHCs are government owned, and LGAs are responsible for managing them. They provide primary care for patients with typically acute conditions, ante-natal services and immunization for children. PHCs are typically staffed by a range of nurses, healthcare assistants and community health extension workers (usually 4–6 staff per PHC). The secondary health-care centres are mostly government-owned and the others are either privately-owned or run by missionary organizations. Cross River State has a tropical climate, with heavy rainfall during the wet season (April—November), and consists of the mangrove, tropical rain forest in the south and central zones, with the savannah woodlands in the north. It has a total land area of about 23,000 km^2^. The vectors of dengue–*Aedes aegypti* and *Aedes albopictus–*have been identified in parts of Cross River from a previous entomological survey [15].

### Study design and sample size

This cross-sectional facility survey was conducted between January and August 2017 in health facilities (mostly PHCs) across Cross River State. It was not possible to obtain a probability sample because of the need to recruit patients presenting at facilities with fever. We therefore used random sampling methods to first select a probability sample of health facilities, but then had to take a non-probability sample of patients from those health centres. Specifically, in the first stage of sampling 10 LGAs were selected with probability proportional to the number of health centres across the three districts. In the second stage of sampling 1 health centre per LGA was randomly selected. Field teams then visited the health centre, initially for 2–3 days, and consecutively recruited patients into the study with the aim of reaching the sample size per facility in all facilities. However, in a number of facilities recruitment was much slower than anticipated. Therefore, in those facilities where recruitment was faster more patients were recruited than the necessary number per facility to reach the overall sample size, and in facilities where recruitment was very low another health centre from the same LGA was randomly selected and patients sampled there as well.

Based on our resources we estimated that we could recruit a total of 396 participants (on average 33 per health centre), which we calculated would allow us to estimate the prevalence of DENV infections with a precision (i.e. 95% confidence interval) of at most ± 7.8%. This made the conservative assumption that we would be estimating a prevalence of 50%, and that there would be a design effect of 2.5, which was a conservative inflation of the design effect found in the Nigeria 2015 Malaria Indicator Survey for the estimated prevalence of malaria in children (aged 6–59 months) based on rapid diagnosis testing [16].

### Study population

The study population was consenting febrile patients presenting to sampled health centres who met the following eligibility criteria: age ≥1 year and fever (axillary temperature of ≥ 37.5°C) for< 10 days.

### Laboratory methods

Following strict aseptic technique and using sterile needles and 10 ml syringes, 6ml of blood was withdrawn by venepuncture. Five mls of blood was delivered into a plain serum tube while 1 ml was put in an EDTA bottle, both labelled with patient hospital and study numbers. Thick blood smears were made on two grease-free slides and stained with 10% Giemsa for 10 minutes. The films were viewed using a compound light transmission microscope at a magnification of X1000. In this study, positive slides were determined by the presence of merozoites or ring forms of the malaria parasites. No schizonts or gametocytes were seen in this study. Two hundred fields were viewed before a film was declared negative.

Blood samples were transported in a cold chain to the University of Calabar Teaching Hospital (UCTH) for serum separation and storage. Serum was obtained from the plain bottle sample by centrifugation at 5000 rpm for 10 minutes, and was used for the rapid detection of DENV immunoglobulin M (IgM)/immunoglobulin G (IgG) antibody using *Onsite* Duo Dengue Ag-IgG/IgM lateral flow immunoassay cassettes (CTK Biotech Inc, San Diego, USA), according to the manufacturer’s instructions. Samples which tested positive were further confirmed using the *RecombiLISA* Dengue IgM and IgG 96-well enzyme linked immunosorbent assay (ELISA) kits (CTK Biotech Inc, San Diego, USA), according to the manufacturer’s instructions. Detection of IgM alone or an IgM:IgG optical density (OD) ratio ≥1.2 was designated as primary dengue, while the detection of IgG alone or an IgM:IgG OD ratio <1.2 was designated as secondary dengue [17–19].

### Data collection methods, instruments and quality assurance

Data was collected from eligible and consenting participants or their caregivers using a proforma that captured the individual-level variables of interest including age, sex, occupation and use of mosquito preventive measures (insecticide treated nets and indoor residual spraying), home roof type, household location (urban/rural), household water storage and travel history. The information obtained was then entered into a data management system, including restrictions over the type and range of values allowed to reduce data entry errors. Quality control checks were performed on 10% of entered records to reduce the occurrence of entry errors. Also, 5% of the laboratory tests were repeated by an independent pathologist to corroborate the laboratory results obtained.

### Outcomes

The primary outcome was the prevalence of a primary or secondary dengue infection among participants with or without malaria. The secondary outcomes were the prevalence of1) primary or secondary dengue without malaria, 2) primary dengue with malaria, 3) primary dengue without malaria, 4) secondary dengue with malaria, 5) secondary dengue without malaria, 6) malaria with/without primary/secondary dengue, 7) malaria with primary/secondary dengue, and malaria without primary/secondary dengue.

### Statistical analysis

Data were analysed using R’s *survey* package [20–22] commands to allow results to account for the clustering of the study design by health centre, via Taylor Series Linearisation methods [22] for complex survey data analysis. Descriptive statistics (means, SDs and percentages) were calculated to describe the characteristics of the sampled population in terms of their general sociodemographic features and known risk factors for dengue (e.g. residence location, dwelling characteristics etc). Percentage and frequency estimates for all outcomes were calculated along with their 95% confidence intervals. Because all outcomes were binary their 95% confidence intervals were calculated using the *survey* package svyciprop function’s logit method, which “fits a logistic regression model and computes a Wald-type interval on the log-odds scale, which is then transformed to the probability scale”. Multivariate logistic regression was also used to evaluate the direction, magnitude and statistical significance of associations between a range of risk/protective factors and dengue (primary or secondary) positivity (with or without malaria co-infection), via adjusted odds ratios and their 95% confidence intervals, with two-sided hypothesis testing conducted at the 5% level. All independent variables were chosen before constructing the model based on evidence from the literature. Observations with missing outcome and/or covariate data were omitted from the model. Given the small size of the dataset this analysis should be treated as strictly exploratory.

### Ethical considerations

Ethical clearance was duly obtained from the Health Research and Ethics Committee of the UCTH with number UCTH/HREC/33/324. Written informed consent was obtained from either the participants or their caregivers (in the case of minors) prior to the recruitment and sample collection. Participation was voluntary and the cost of the tests were not be borne by the participants.

## Results

A total of 11 health centres were sampled, with an additional health centre selected in one LGA due to a very low recruitment rate. Dates of patient recruitment varied across health centres. Across all health centres the earliest date of patient recruitment was 4 Jan 2017 and the latest was 24 Aug 2017. Across all health centres the range in the number of days between first and final patient recruitment dates was 0–232 (median = 97).There was also a large amount of variation in the number of patients recruited per facility, with a median sample size of 34 and a range of 2-96.There were a total of 420 participants with a mean age of 35 and 63% being female(Table 1).Twenty-six percent (26%) had university education, while 17% had not received any formal education. Fifty-nine (59%) resided in a rural area, with the mean household size being 5. Most (90%) participants lived in houses roofed with sheet metal. 46% had waste around their homes, but 86% also indicated that they practiced some form of environmental management to keep waste down to a minimum. Forty-nine (49%) reported sleeping under a mosquito net during the previous week (Table 2). The use of mosquito nets on doorways and windows was a common practice (65%). The use of antimalarials prior to presentation at a healthcare facility was also frequently reported (44%). The average axillary temperature among participants was 38°C, and the mean duration of fever was 8 days. Many of the participants presented with symptoms indicative of urinary tract and respiratory infections, namely dysuria, cough/coryza, breathlessness and sore throats (Table 1).

**Table 1:** Study population characteristics.

Total N		420
Age		35 (±19)
Sex	F	63% (263)
	M	37%(157)
Educational level	None	17% (72)
	Primary	28% (115)
	Secondary	29% (122)
	Tertiary	26% (109)
	NA	0% (2)
Axillary temperature		≥38.1 (±0.72)
Days fever present prior to arrival		8(±12)
Other family members with fever	No	58%(243)
	Yes	42%(176)
	NA	0%(1)
Cough/cold	No	51%(215)
	Yes	49%(205)
Difficulty in breathing	No	76%(318)
	Yes	24%(101)
	NA	0%(1)
Dysuria	No	77%(324)
	Yes	23%(95)
	NA	0%(1)
Sore ear	No	89%(372%)
	Yes	11%(44)
	NA	1%(4)
Sore throat	No	80%(335)
	Yes	20%(84)
	NA	0%(1)
Rash	No	84%(351)
	Yes	16%(67)
	NA	0%(2)
Tender lymphadenopathy	No	89%(372)
	Yes	11%(46)
	NA	0%(2)

Data are n, mean (SD) or %(n). For categorical variables missing data frequencies are provided but all other category frequencies are calculated excluding missing data

**Table 2:** Details of housing conditions and protection against malaria.

Total N		420
Household location	Urban	41%(171)
	Rural	59%(249)
Household size		5(±3)
Number of bedrooms		3(±3)
Home roof type	Sheet metal	90%(377)
	Grass thatched	5%(20)
	Tiles	4%(17)
	Other	1%(3)
	NA	1%(3)
Presences of waste	No	54%(224)
	Yes	46%(189)
	NA	2%(7)
Presence of water storage	No	24%(102)
	Yes	76%(317)
	NA	0%(1)
Environmental management	No	14%(59)
	Yes	86%(357)
	NA	1%(4)
Slept under treated net in the previous week	No	51%(211)
	Yes	49%(204)
	NA	1%(5)
Use mosquito net screen	No	35%(147)
	Yes	65%(273)
Use indoor spray coil	No	58%(241)
	Yes	42%(173)
	NA	1%(6)
Apply insect repellent	No	95%(396)
	Yes	5%(22)
	NA	0%(2)
Taken anti-malarial medication during present illness	No	56%(223)
	Yes	44%(178%)
	NA	5%(19)

Data are n, mean (SD) or %(n). For categorical variables missing data frequencies are provided but all other category frequencies are calculated excluding missing data

6% (95% CI: 2%, 13%) of participants had antibody test results consistent with either a primary (first infection) or secondary dengue infection with or without malaria, and 4% (95% CI: 2%, 9%) were positive for primary/secondary dengue alone (i.e. without malaria). Fifty percent (95% CI: 44%, 57%) of participants who had a positive malaria test were negative for both primary and secondary dengue, and 52% (95% CI: 46%, 58%) of the participants were positive for both malaria and primary/secondary dengue (Table 3).

**Table 3:** Dengue and malaria test outcomes.

Test diagnosis	n (N = 420)	% (95% CI)
Primary/secondary dengue with/without malaria	24	6% (2%, 13%)
Primary/secondary dengue without malaria	16	4% (2%, 9%)
Primary dengue with malaria	7	2% (1%, 5%)
Primary dengue without malaria	8	2% (1%, 6%)
Secondary dengue with malaria	1	0% (0%, 2%)
Secondary dengue without malaria	8	2% (1%, 5%)
Malaria with/without primary/secondary dengue	218	52% (46%, 58%)
Malaria with primary/secondary dengue	8	2% (1%, 6%)
Malaria without primary/secondary dengue	210	50% (44%, 57%)

The logistic regression model showed that age, gender, educational level, place of residence, presence of waste around house and the use of any household malaria protection were not clearly associated with having either primary and/or secondary dengue (Table 4). Household roof type was originally included in the model as a known risk factor for dengue, but due to complete separation for some categories had to be removed.

**Table 4:** Associations between dengue diagnosis (primary/secondary) and patient characteristics.

Outcome	% (95% CI); p-value^a^
Age	1 (1–1); 0.35
Male (ref = female)	0.6 (0.2–1.8); 0.39
Secondary/higher education (ref = none/primary)	1 (0.6–1.7); 0.97
Rural household (ref = urban)	1.1 (0.4–1.7); 0.84
Waste present at household (ref = no)	0.5 (0.2–1.4); 0.29
Water storage present at household (ref = no)	0.9 (0.3–2.6); 0.89
Any household mosquito protection used^b^ (ref = no household mosquito protection used)	0.6 (0.3–1.5); 0.38

9 (2.1%) observations were omitted due to missing covariate data.

^a^Coefficients were obtained from a logistic regression with the outcome of having a dengue (primary/secondary) diagnosis vs having no dengue diagnosis, and were converted to odds ratios via exponentiation.

^b^Any mosquito household protection used included either a mosquito net screen, and/or a treated bed net and/or an indoor spray coil.

## Discussion

We found that 6% of patients tested positive for primary or secondary DENV infections either with or without a malaria co-infection. In these undifferentiated fever cases 50% tested positive for malaria but not dengue, while just 2% had co-existing primary dengue and malaria infections (and none had secondary dengue and malaria positivity).Symptoms indicative of urinary tract and respiratory infections were commonplace among this cohort and may account for some proportion of the fevers experienced. Dengue seroprevalence from a survey done in Maiduguri [11] Northern Nigeria was 10.1% (testing specifically for DEN V 3 using a microneutralization assay) and was 17.2% in Ogbomoso [13], South Western Nigeria (testing for dengue IgM using ELISA). However, these studies used a one-stage testing for dengue antibodies using microneutralization and ELISA respectively. To the best of our knowledge, this is the first survey of dengue seroprevalence from any setting in the South Eastern part of Nigeria.

Nigeria is a West African country in which dengue is reported to be endemic [11]. However, it is likely that many cases of dengue in Nigeria are often undiagnosed or misdiagnosed as malaria or referred to as fever of unknown cause. Although there have been several reported isolated outbreaks of dengue infection, still it is likely that many outbreaks have been neglected, un recognized and under-reported due to unavailability of diagnostic tools and staff unawareness in health institutions [12]. A clear method of identifying dengue infection from among other acute undifferentiated tropical febrile illnesses is vital to facilitate appropriate triage of patients and better clinical management of dengue cases. Serologic tests for dengue are relatively inexpensive, quick and easy to perform and are available as point-of-care tests which detect dengue NS1 antigen and anti-dengue IgM/IgG antigens. Because healthcare facilities in many dengue endemic countries lack laboratory support, such simple diagnostic tests are desirable. We used a lateral-flow diagnostic assay which cost less than $2 per test and could be done by laboratory technicians at primary care level. This point-of-care lateral flow assay for dengue has been validated for use in dengue endemic areas [23,24] as it fulfils the World Health Organization (WHO) Affordable, Sensitive, Specific, User-friendly, Rapid & Robust, Equipment-free, and Delivered (ASSURED) criteria for point-of-care testing [25].

Dengue IgM levels begin to rise by the third day of a primary infection and peak at 2 weeks after the onset of fever and may remain detectable for up to 6 months or longer following disease resolution. IgG is detectable at the end of the first week of illness and can persist for life. ELISA tests can assay for IgM and IgG levels and the IgM: IgG ratio is useful in distinguishing primary from secondary dengue virus infections. An IgM: IgG ratio of ≥ 1.2 is indicative of primary dengue infection while an IgM: IgG ratios of < 1.2 is indicative of secondary dengue infection [18].

The diagnosis of dengue is further complicated by malaria co-infection as demonstrated in our study. A recent survey done in the inland western Nigerian city of Ibadan put the number of persons with active dengue infection among confirmed malaria cases at 10%. Among this cohort of malaria cases, all of them were found to be positive for dengue IgG antibodies suggestive of a past dengue infection and consistent with endemicity of dengue virus in this area [26]. All these reflect a significant public health challenge that needs to be prioritised.

The use of mosquito nets has been recognised to be the most effective strategy for malaria control [27], but the use of mosquito treated nets among this cohort did not meet the targets set for malaria control in an endemic area. Effective use of the long-lasting insecticide treated nets (LLIN) has been shown to significantly reduce the transmission of *Aedes*-borne diseases such as dengue, Zika, yellow fever and chikungunya [28]. Interestingly, Aedes aegypti density has been shown by the use of LLIN as window curtains [29]. Therefore, the LLIN appears to hold much promise for the control of the diseases transmitted by these endophilic mosquitoes and its widespread use should be promoted in the relevant climes. A significant proportion (44%) of our participants took antimalarial treatment without malaria testing as recommended in malaria management guidelines [30]. Up to 70% of all febrile cases in Nigeria are believed to be misdiagnosed and presumptively treated as malaria. The erroneous belief that malaria accounts for virtually all cases of fever in Nigeria is widespread among the populace. It is commonplace for persons with fever in Nigeria to self-medicate with two or three antimalarial drugs before presenting to a health facility [31]. This is consistent with the findings here. Following improvements in malaria control efforts in low-middle income country (LMIC) contexts, it is believed that bacterial and viral pathogens account for the majority of cases of acute febrile illnesses [32,33]. However, there appears to be limited baseline understanding of these pathogens with a disproportionate focus on malaria leading to misdiagnosis and unwarranted treatment. This may result in a misapplication of scarce resources and inadvertently drive resistance to the currently available antimalarials. Indeed the preoccupation with malaria in the LMICs may be constituting a barrier to understanding the complex communicable diseases epidemiology that characterise these countries.

Rapid population growth, unplanned urbanization, increased international travel, agricultural development, possible global climate changes are some of the factors that have been put forward to explain the extensive transmission of dengue in these areas [34]. Other enabling factors include ineffective mosquito control measures and the limited allocation of resources to public health infrastructure [35]. However, none of the variables in our logistic regression model (namely age, sex, education, place of residence, presence of waste around house, presence of water storage at house, and the use of any household malaria prevention strategies) were associated with dengue infection status. It is not clear why no associations were found, but limited sample size and therefore power may be a major reason.

Clearly there is need it for algorithmic guidelines, including universal malaria and selective dengue testing, for the diagnosis and management of patients with fever presenting to primary and secondary care in Nigeria. This pragmatic framework for triage and testing of undifferentiated cases of fever in resource limited settings is critical in determining the clinical outcomes of patients with dengue. These guidelines will need to reflect the fact that about half of these undifferentiated cases are neither due to malaria nor dengue, but may rather be related to respiratory or urinary infections. Such guidelines will need to include methods of identifying dengue related complications to promote optimum management and referral of relevant cases.

Our study confirms the presence of DENV infections in patients attending PHCs in this south eastern province of Nigeria. Surveillance for dengue is vital in this setting for identifying outbreaks and initiating an early response.

This study has several limitations. First, our survey did not use a probability sampling method, given that although we took a probability sample of health facilities we had to take a non-probability sample of patients from within those facilities. This therefore prevented the calculation of weights and limits the robustness and generalisability of the estimates, given that inferential statistics formally assume the data come from a random probability sample. Second, this is a facility-based survey, and so we cannot generalize our results to the community or wider population outside of those attending health facilities for fever, which would require a population survey. Third, dengue shows seasonal trends, and the data were collected over a large proportion of the year and importantly with uneven effort across that time period. Hence, the overall percent prevalence estimates do not represent the prevalence from any single part of the year, nor do they represent an evenly sampled average across the whole year. The results should therefore be treated cautiously as likely indicative of the broad level of dengue infections in patients attending PHCs with fever. Fourth, we were unable to definitively determine the proportion of patients with other febrile conditions.

## Supporting information

S1 Table Checklist: STROBE Checklist. (DOC)

S2 Table Dataset. (SAV)
